# Monitoring the Invasion of *Spartina alterniflora* Using Very High Resolution Unmanned Aerial Vehicle Imagery in Beihai, Guangxi (China)

**DOI:** 10.1155/2014/638296

**Published:** 2014-05-04

**Authors:** Huawei Wan, Qiao Wang, Dong Jiang, Jingying Fu, Yipeng Yang, Xiaoman Liu

**Affiliations:** ^1^Satellite Environmental Application Center, Ministry of Environmental Protection, Beijing 100094, China; ^2^State Key Laboratory of Resources and Environmental Information System, Institute of Geographic Sciences and Natural Resources Research, Chinese Academy of Sciences, Beijing 100101, China; ^3^University of Chinese Academy of Sciences, Beijing 100049, China

## Abstract

*Spartina alterniflora* was introduced to Beihai, Guangxi (China), for ecological engineering purposes in 1979. However, the exceptional adaptability and reproductive ability of this species have led to its extensive dispersal into other habitats, where it has had a negative impact on native species and threatens the local mangrove and mudflat ecosystems. To obtain the distribution and spread of *Spartina alterniflora*, we collected HJ-1 CCD imagery from 2009 and 2011 and very high resolution (VHR) imagery from the unmanned aerial vehicle (UAV). The invasion area of *Spartina alterniflora* was 357.2 ha in 2011, which increased by 19.07% compared with the area in 2009. A field survey was conducted for verification and the total accuracy was 94.0%. The results of this paper show that VHR imagery can provide details on distribution, progress, and early detection of *Spartina alterniflora* invasion. OBIA, object based image analysis for remote sensing (RS) detection method, can enable control measures to be more effective, accurate, and less expensive than a field survey of the invasive population.

## 1. Introduction

*Spartina alterniflora (S. alterniflora) *is a perennial rhizomatous C4 grass of Poaceae that was originally found in the intertidal zones on the Atlantic coastal areas of North America.* S. alterniflora* spreads through clonal propagation by rhizome and sexual reproduction by seed, and thus it is a rapidly spreading plant [1].* S. alterniflora* has numerous biological traits, such as fast growth, great productivity, high tolerance to salt, and a well-developed belowground system that make it a typical ecosystem engineer [2, 3].* S. alterniflora* was introduced to China for ecological engineering purposes in December 1979 [4]. However, the exceptional adaptability and reproductive ability of this species have led to its extensive dispersal into habitats, where it has had a negative impact on native species [5–8]. It is on the first list of 16 invasive species issued by the State Environmental Protection Administration of China in 2003.* S. alterniflora *is now widely distributed along the east coast of China, from Tianjin to Beihai, Guangxi [9]. In the Guangxi province,* S. alterniflora* was planted in Dandou, Beihai, Dangjiang, and Hepu in 1979 [10], and it then expanded rapidly and now threatens the local mangrove and mudflat ecosystems. Therefore, it is very important to determine the distribution of* S. alterniflora* to support its elimination and to conserve biodiversity [11–13].

It is difficult to obtain a detailed distribution map of* S. alterniflora* by the traditional ground survey method because the species usually grows in the intertidal zone. Remote sensing technology provides a viable means of obtaining overall and accurate information sources of land cover and composition and has been widely applied to monitoring invasive species, vegetation dynamics, and biodiversity conservation since the 1980s [2, 5, 6, 14–16]. RS of herb species is possible only if the data provides an adequate amount of spectral and/or spatial detail; the species is distinct from the surrounding species and background; it forms dense and uniform stands and/or it is large enough to be detected [17–20]. There exists a growing body of work on the monitoring of* S. alterniflora *using satellite imagery [16, 21–23] and hyperspectral imagery [24–27]. However, very high spatial resolution UAV imagery applications focusing on* S. alterniflora* invasions are not common. The resolution of moderate spatial satellite imagery is relatively low for determining an accurate distribution of* S. alterniflora*, while the resolution of spatial hyperspectral imagery is very poor and usually lacks coverage. This study uses color from very high spatial resolution UAV imagery to identify invasive* S. alterniflora *by its spatial and spectral characteristics and background. UAV imagery has the advantage of measuring at a very high spatial resolution within 1 meter, enabling the collection of data at a specific time and area on demand.

In previous studies dealing with RS mapping of invasive plants, manual interpretation methods were used the most, which is laborious, time-consuming, and not feasible for a large area [18, 28]. Therefore, it is important to develop automated or semiautomated image processing techniques to classify VHR imagery. Object-based classification is a relatively recent technology in which textural and shape information is used in addition to spectral information for classification, which allows for identification of individual objects as opposed to single pixels. Pixels are aggregated into image objects by segmentation, which is defined as the division of RS images into objects that are homogenous with regard to spatial and/or spectral characteristics [29, 30]. Few studies have used object-based image analysis (OBIA) for herbaceous or invasive species classification [17, 31, 32]. Franklin et al. [7] found that the incorporation of texture in addition to spectral information increased classification accuracy by 10%–15%. The development of OBIA has promoted the possibility of automated or semiautomated processing of VHR imagery.

The area covered by* S. alterniflora* in Guangxi is 251 ha, representing only 0.74% of the total area of* S. alterniflora* in China [1]. However, there are three ecologically sensitive targets in the study area, namely, two National Nature Reserves and a scenic locale. Therefore, it is essential to map the spatial distribution of* S. alterniflora *to analyze its ecological effects. The objectives of this study are the following: (1) to propose an object-based image analysis (OBIA) identification method for invasive* S. alterniflora* based on UVA imagery and verify the results using field survey data and (2) to analyze the spatial distribution of* S. alterniflora* in 2011 in Beihai, in the Guangxi province of China.

## 2. Study Area

**Figure 1:**
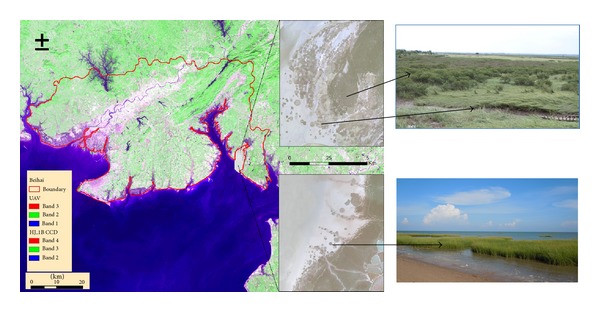
Study area.

Beihai is a prefecture-level city in the southern part of Guangxi, China, located at the longitudes of 108°50′45′′~109°47′28′′ and the latitudes of 20°26′~21°55′34′′ at the southern tip of the Gulf of Tonkin. The name of the city means “north of the sea” in Chinese, signifying its status as a seaport and granting it historical importance as a port of international trade. With the sea on three sides, the total length of the coastline of Beihai is over 500 km. Beihai has a monsoon-influenced humid subtropical climate, with mild to warm winters, long, hot (but not to the extremes) summers, and very humid conditions year-round. Rain is both the heaviest and most frequent from June to September. The area receives approximately 2,000 hours of sunshine annually. The location of the study area is shown in Figure 1.

## 3. Data and Methods

### 3.1. Data Collection

To fully understand the distribution of* S. alterniflora* in Beihai, Guangxi, we selected the entire Beihai Administrative district as the study area and applied remote sensing data, including the HJ-1 CCD, UAV, and other auxiliary data. HJ-1 satellites were launched in September 2008 and were placed into service in March 2009. The satellites are now operating normally, playing an important role in China's ecological, environmental, and disaster monitoring [33]. The results obtained from HJ-1 can be served as a background to reveal the spread tendency and speed from 2009 to 2011. The VHR imagery was collected from Sep. 6, 2011, to Oct. 2, 2011, by the unmanned aerial vehicle, which carried Cannon 5D cameras at the height of 800 meters. The flight route was along the coastal zone where the* S. alterniflora* invasion is very problematic, including the concentrated Dandou Sea and Shatian port.

The details of the collected data are listed in Table 1.

### 3.2. Data Processing

In this study, the spatial distribution of* S. alterniflora* in 2009 was mapped by HJ-1 CCD imagery by manual interpretation with the supporting of substantive field survey data. OBIA was used to distinguish* S. alterniflora* from mangroves in 2011. In OBIA, the image is segmented into groups of contiguous pixels (image objects) that are subsequently classified according to texture, context, spatial relationship, and other characteristics which contain both spectral and spatial information. OBIA was conducted according to the following steps.

**Step 1 (multiresolution segmentation)**

**Figure 2:**
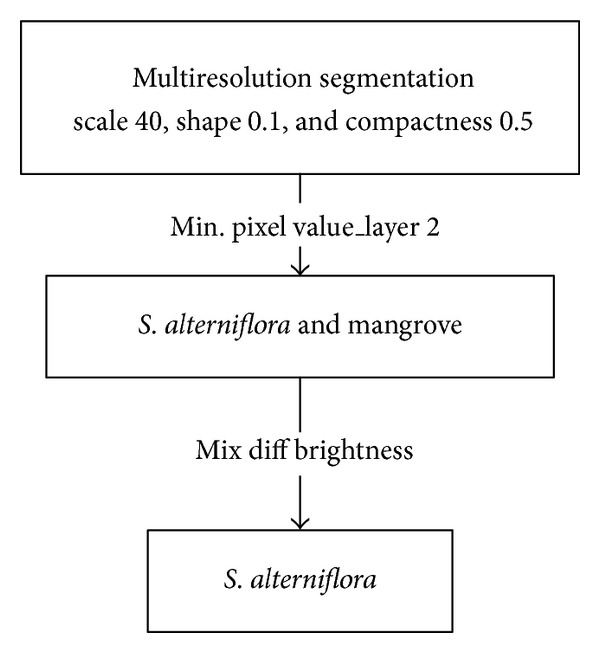
OBIA classification scheme for* S. alterniflora.*

Multiresolution image segmentation is a local optimization process which creates groups of homogeneous pixels (image objects) that are subsequently classified according to different space and spectral features between objects. After testing, the simplest thresholding segmentation method was adopted based on the obvious difference between* S. alterniflora* and mangroves. Several scales were tested, and segmentation outputs were evaluated. The best parameters to extract the* S. alterniflora* were identified (see determination in Figure 2).

**Step 2 (construction of feature indices)**

The features used to extract the targets of interest usually include the characteristics related to spectrum (brightness, mean layer, maximum pixel values, standard deviation, hue, saturation, and intensity transformation for RGB), shape (area and length/width), texture (compactness, GLCM homogeneity, GLCM dissimilarity, and GLCM contrast), and context (existence of thematic layer, relative border, contrast and edge contrast of neighbor pixels, existence of superobject, and growth region in certain conditions) [17]. In this paper, a variable control method is used to select reasonable segmentation parameters. The values of segmentation parameters are as follows: segmentation scale 40, shape index parameter 0.1, and compactness index weight 0.5.

**Step 3 (determination of classification threshold)**

Rules based on the classification of VHR were applied on the segmented image objects using the feature indices chosen in the second step. Training sites are identified based on the field survey pictures and local knowledge provided by the expert. Thresholds for the indices used for classification were estimated from the training data.

**Step 4 (verification and accuracy assessment)**

The classification result was verified by the field survey samples. The error rate was calculated in three typical regions where* S. alterniflora *was the most concentrated (see Figure 4, the location of Dandou Sea and Shatian port). There were 72, 64, and 30 samples obtained from the northwest of Dandou Sea, southeast of Dandou Sea, and Shatian port, respectively.

## 4. Results and Analysis

### 4.1. VHR Based Identification of* S. alterniflora*

#### 4.1.1. Segmentation and Feature Analysis

**Figure 3:**
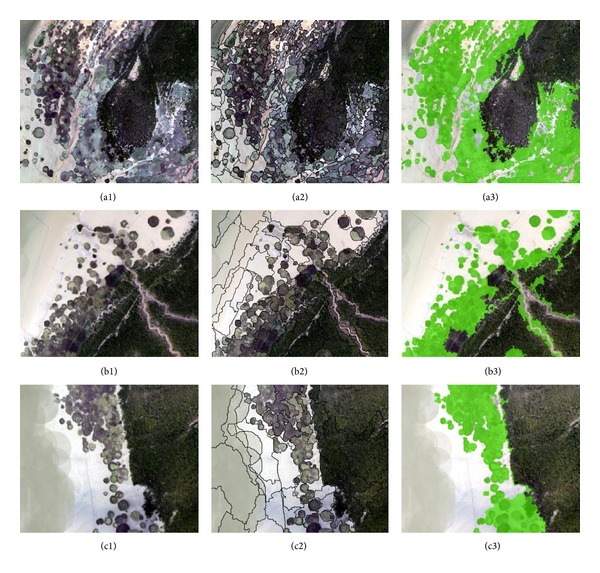
Three typical regions from north of Dandou Sea ((a1) to (a3)), south of Dandou Sea ((b1) to (b3)), and Shatian port ((c1) to (c3)) were selected. (a1)–(c1) shows the original VHR image; the segmentation parameters of the three parts are scale 40, shape 0.1, and compactness 0.5 and the results are (a2)–(c2); the features for extracting* S. alterniflora* from (a3) and (c3) are “Mix diff ≤ 0.09, brightness: 105–136” and the features for extracting* S. alterniflora* from (b3) are “Mix diff ≤ 0.08, brightness ≤ 136.”

The most concentrated areas of* S. alterniflora *are around the Dandou Sea and Shatian port where the UAV cover. We divided the study area into three parts because of the restriction on data processing. The three parts were divided corresponding to the field survey sites. To show the details of classification, three typical regions were selected from each of the three parts, which includes northwest of Dandou Sea, southeast of Dandou Sea, and Shatian port (see Figure 3).

#### 4.1.2. Results of Classification

Based on the monitoring results, we found the following.

**Figure 4:**
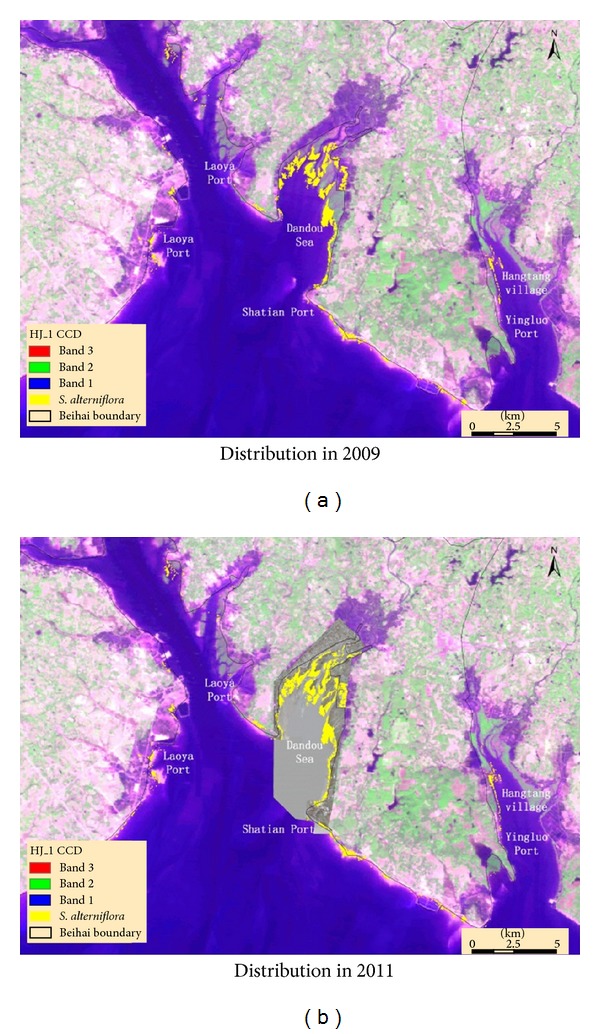
The spatial distribution of* S. alterniflora.*

**Figure 5:**
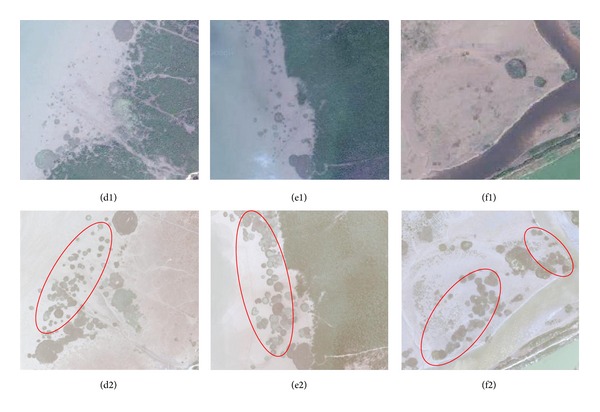
(d1)–(f1) are maps obtained from Google Earth in 2009 of the three regions of northwest of Dandou Sea, southeast of Dandou Sea and Shatian port. (d2)–(f2) are the same regions corresponding to (d1)–(f1) obtained from the UVA imagery with the expanded* S. alterniflora *denoted by red circle.

1. In 2011, the area occupied by* S. alterniflora* is 357.2 ha, mainly distributed in the 149 km coastal beach located east of Beihai. The length of the coastal zone that this species has invaded is 25% of the entire coastal zone. Figure 4 shows its detailed distribution in Beihai.
2. More than 70% of the species is concentrated in Dandou Sea. Other relatively concentrated regions include Haitang village, Laoya port, and Danchang.
3. Compared with the monitoring results of 2009 (300 ha), the* S. alterniflora* distribution area increased by 57.2 ha (19.07%). The expansion trend was obvious, especially in east-to-west (Beihai) direction and in south-to-north direction of Laoya port, Yingluo port, and Haitang Village.  Figure 5 shows images using contrast to denote the expanded* S. alterniflora*.

### 4.2. Accuracy Assessment

To verify the precision of the extractions, the extraction results were compared to sample points that were conducted by the field survey. The samples were distributed evenly around the Dandou Sea, corresponding to the UAV imagery in September 2011. A total of 166 samples were determined in the research area, including 72, 64, and 30 samples in the north of Dandou Sea, south of Dandou Sea, and Shatian port, respectively. Of the 166 samples, the results for the north of Dandou Sea are correct for 67 points, with a precision of 93.1%; for the south of Dandou Sea, the results are correct for 60 points, with a precision of 93.7%; and for the Shatian port region, which is relatively small, the results are correct for 29 points, with a precision of 96.7%. For the total area, the accuracy has reached 94.0%, which indicates that the OBIA-based VHR classification scheme is an effective and accurate methodology.

### 4.3. Impacts on the Ecological Environment

#### 4.3.1. Threats to the Ecologically Sensitive Targets in the Coastal Area of Beihai

There are three ecologically sensitive targets in the study area, including the Shankou Mangrove National Nature Reserve, Hepu Dugong National Nature Reserve, and Yintan scenic spot. The results show that the Shankou Mangrove National Nature Reserve has been seriously affected by the invasion of* S. alterniflora*. There is an invasion area of 1.53 ha in the core protected area, 87.13 ha in the buffer area, and 34.87 ha in the test area. The invasion of* S. alterniflora* poses a great threat to the ecological safety of mangroves. The distance of the* S. alterniflora *from the western coastal line to the Yintan scenic locale is only 8.83 km, representing another potential threat to the ecological health of the area.

#### 4.3.2. The Potential Impact on the Ecological Security of the Coastal Zones

The greater majority of* S. alterniflora *is now in Beihai, which is located in the eastern coast, and has been a threat to the ecological sensitive targets in the coastal zone. However, there has not been any serious ecological damage. According to the monitoring results,* S. alterniflora* has a tendency to spread westward. If* S. alterniflora* spreads to the western coastal zone of Beihai, it may spread to the Dafeng Estuary, Maowei Estuary, the East Bay and West Bay of Fangcheng city, Beilun Estuary, and, finally, Vietnam. The developed estuaries, shoals of silt, low salinity sea water, and rich nutrients in the areas mentioned all aid in the growth and spread of* S. alterniflora*. This situation poses a serious threat to the marine biodiversity, mudflat aquaculture, fisheries, and the navigable channels in the seas west of Beihai, potentially affecting the development of the port industry or possibly even resulting in environmental diplomacy problems.

## 5. Conclusions

This study used UVA and HJ-1 CCD imagery to map* S. alterniflora*, an invasive herb species in the Beihai coastal zone in Guangxi, China. Based on the remote-sensing materials and methods used in this study, the following conclusions were obtained.

In the first instance, the spatial distribution of* S. alterniflora* in 2009 was mapped by HJ-1 CCD imagery by manual interpretation. The invasion area is approximately 300 ha, posing a great threat to the Shankou Mangrove National Nature Reserve, Hepu Dugong National Nature Reserve, and Yintan scenic locale.

Moreover, in 2011, the details of invasive* S. alterniflora* were identified by the UVA imagery based on OBIA. The coverage area of* S. alterniflora *in 2011 is 357.2 ha, which is 19.07% greater compared with the area in 2009.* S. alterniflora* is mainly distributed in the east coast from the Xicun port to the Yingluo port and has a tendency to spread westward. The invasion of* S. alterniflora* has the potential to threaten the navigable channels from Beihai to Fangcheng and even to Vietnam if the invasion cannot be controlled effectively.

Furthermore, a field survey was conducted for verification. The OBIA classification has proven to be an effective and accurate method for VHR imagery. The accuracy of the classification performance reached 94.0%, indicating that the methodology described in our study is potentially applicable to other similar areas and/or other invasive species.

Finally, in our study, the VHR imagery can provide details on the distribution, progress, and early detection of the spreading of* S. alterniflora*. The OBIA-based RS detection method applied in this paper is a more effective, more accurate, and less expensive control measure in plant invasion monitoring.

## Figures and Tables

**Table 1:** Data Information.

Data	Spatial region	Spatial resolution (m)	Imaging date	Spectral resolution (µm)
HJ-1 CCD	Beihai	30	Sep. 11, 2009–Nov. 30, 2011	Band 1: 0.43–0.52 Band 2: 0.52–0.60 Band 3: 0.63–0.69 Band 4: 0.76–0.90

UAV imagery	Coastal areas	0.8	Sep. 6, 2011–Oct. 2, 2011	Color orthophoto

## References

[1] Lu J, Zhang Y (2013). Spatial distribution of an invasive plant *Spartina alterniflora* and its potential as biofuels in China. *Ecological Engineering*.

[2] Schmidt KS, Skidmore AK (2003). Spectral discrimination of vegetation types in a coastal wetland. *Remote Sensing of Environment*.

[3] Li B, Liao C-H, Zhang X-D (2009). *Spartina alterniflora* invasions in the Yangtze River estuary, China: an overview of current status and ecosystem effects. *Ecological Engineering*.

[4] Chung C-H (2006). Forty years of ecological engineering with *Spartina* plantations in China. *Ecological Engineering*.

[5] Huang CY, Asner GP (2009). Applications of remote sensing to alien invasive plant studies. *Sensors*.

[6] Zuo P, Zhao S, Liu C, Wang C, Liang Y (2012). Distribution of *Spartina* spp. along China’s coast. *Ecological Engineering*.

[7] Franklin SE, Hall RJ, Moskal LM, Maudie AJ, Lavigne MB (2000). Incorporating texture into classification of forest species composition from airborne multispectral images. *International Journal of Remote Sensing*.

[8] Rosso PH, Ustin SL, Hastings A (2006). Use of lidar to study changes associated with *Spartina* invasion in San Francisco Bay marshes. *Remote Sensing of Environment*.

[9] Wang Q, An S-Q, Ma Z-J, Zhao B, Chen J-K, Li B (2006). Invasive *Spartina alterniflora*: biology, ecology and management. *Acta Phytotaxonomica Sinica*.

[10] Mo Z, Fan H, Liu L (2010). Investigation on smooth Cordgrass (*Spartina alterniflora*) along Guangxi coastal tidal zone. *Guangxi Sciences*.

[11] Li H, Zhang L (2008). An experimental study on physical controls of an exotic plant *Spartina alterniflora* in Shanghai, China. *Ecological Engineering*.

[12] Yuan L, Zhang L, Xiao D, Huang H (2011). The application of cutting plus waterlogging to control *Spartina alterniflora* on saltmarshes in the Yangtze Estuary, China. *Estuarine, Coastal and Shelf Science*.

[13] Ge Z, Cao H, Zhang L (2013). A process-based grid model for the simulation of range expansion of *Spartina alterniflora* on the coastal saltmarshes in the Yangtze Estuary. *Ecological Engineering*.

[14] Zhang WL, Zeng C, Tong C, Zhang Z, Huang J Analysis of the expanding process of the Spartina alterniflora salt marsh in Shanyutan Wetland, Minjiang River estuary by remote sensing.

[15] Laba M, Downs R, Smith S (2008). Mapping invasive wetland plants in the Hudson River National Estuarine Research Reserve using quickbird satellite imagery. *Remote Sensing of Environment*.

[16] Ayres DR, Smith DL, Zaremba K, Klohr S, Strong DR (2004). Spread of exotic cordgrasses and hybrids (*Spartina* sp.) in the tidal marshes of San Francisco Bay, California, USA. *Biological Invasions*.

[17] Mullerova J, Pergl J, Pysek P (2013). Remote sensing as a tool for monitoring plant invasions: testing the effects of data resolution and image classification approach on the detection of a model plant species *Heracleum mantegazzianum* (giant hogweed). *International Journal of Applied Earth Observation and Geoinformation*.

[18] Müllerová J, Pyšek P, Jarošík V, Pergl J (2005). Aerial photographs as a tool for assessing the regional dynamics of the invasive plant species *Heracleum mantegazzianum*. *Journal of Applied Ecology*.

[19] Bradley BA, Mustard JF (2006). Characterizing the landscape dynamics of an invasive plant and risk of invasion using remote sensing. *Ecological Applications*.

[20] Tóth JP, Varga K, Végvári Z, Varga Z (2013). Distribution of the Eastern knapweed fritillary (*Melitaea ornata* Christoph, 1893) (Lepidoptera: Nymphalidae): past, present and future. *Journal of Insect Conservation*.

[21] Gavier-Pizarro GI, Kuemmerle T, Hoyos LE (2012). Monitoring the invasion of an exotic tree (*Ligustrum lucidum*) from 1983 to 2006 with Landsat TM/ETM + satellite data and Support Vector Machines in Córdoba, Argentina. *Remote Sensing of Environment*.

[22] Huang H, Zhang L (2007). Remote sensing analysis of range expansion of *Spartina alterniflora* at Jiuduansha shoals in Shanghai. China. *Journal of Plant Ecology*.

[23] Varga K, Dévai G, Tóthmérész B (2013). Land use history of a floodplain area during the last 200 years in the Upper-Tisza region (Hungary). *Regional Environmental Change*.

[24] Wang M, Zou Y (2008). Research on spatial distribution of Canada goldenrod based on“ 3S” technology. *Journal of Heilongjiang Institute of Technology*.

[25] Wan H, Wang C, Li Y, Wang Q, Li J, Liu X (2010). Monitoring an invasive plant using hyperspectral remote sensing data. *Transactions of the Chinese Society of Agricultural Engineering*.

[26] Lawrence RL, Wood SD, Sheley RL (2006). Mapping invasive plants using hyperspectral imagery and Breiman Cutler classifications (randomForest). *Remote Sensing of Environment*.

[27] Pengra BW, Johnston CA, Loveland TR (2007). Mapping an invasive plant, *Phragmites australis*, in coastal wetlands using the EO-1 Hyperion hyperspectral sensor. *Remote Sensing of Environment*.

[28] Gergely S, Szilárd S, Nikoletta M, Anita K Accuracy assessment of the ASTER GDEM and the SRTM databases: a case study, Hungary.

[29] Ryherd S, Woodcock C (1996). Combining spectral and texture data in the segmentation of remotely sensed images. *Photogrammetric Engineering and Remote Sensing*.

[30] Laliberte AS, Rango A, Havstad KM (2004). Object-oriented image analysis for mapping shrub encroachment from 1937 to 2003 in southern New Mexico. *Remote Sensing of Environment*.

[31] Walsh SJ, McCleary AL, Mena CF (2008). QuickBird and Hyperion data analysis of an invasive plant species in the Galapagos Islands of Ecuador: implications for control and land use management. *Remote Sensing of Environment*.

[32] Jones D, Pike S, Thomas M, Murphy D (2011). Object-based image analysis for detection of Japanese Knotweed s.l. taxa (polygonaceae) in Wales (UK). *Remote Sensing*.

[33] Wang Q, Wu C, Li Q, Li J (2010). Chinese HJ-1A/B satellites and data characteristics. *Science China Earth Sciences*.

